# Analysis and contrast of psoriasis disease burden trends in China and globally from 1990 to 2021

**DOI:** 10.3389/fpubh.2025.1541292

**Published:** 2025-03-06

**Authors:** Keyi Yu, Cheng Cao, Feilong An, Aie Xu, Xingang Wu

**Affiliations:** ^1^Department of Dermatology, Hangzhou Third Hospital Affiliated to Zhejiang Chinese Medical University, Hangzhou, China; ^2^Department of Dermatology, Hangzhou Third People's Hospital, Hangzhou, China

**Keywords:** psoriasis, trend, incidence, prevalence, disability-adjusted life years, disease burden

## Abstract

**Objective:**

This study aimed to delineate the temporal tendency in the age and gender burden of psoriasis in China, spanning from 1990 to 2021, encompassing metrics such as incidence, prevalence, and disability-adjusted life years (DALYs). Furthermore, it sought to contrast these findings with the global disease burden. It also purposed to assess the impacts of age, time, and birth cohort, as well as to forecast the psoriasis burden in China for the upcoming 15 years.

**Methods:**

Utilizing open-access data from the Global Burden of Disease (GBD) database spanning 1990 to 2021, this study comprehensively examined the burden of psoriasis in China and globally. In China, a detailed analysis was conducted, emphasizing dimensions such as age, gender, and temporal trends. Join-point regression models were employed to calculate the average annual percentage change (AAPC). Furthermore, age-period-cohort (APC) analyses assessed the effects of age, time, and birth cohort, while an extended autoregressive integrated moving average model (ARIMA model) was used to forecast the psoriasis burden in China from 2022 to 2036.

**Results:**

Between 1990 and 2021, China experienced significant changes in its age-standardized incidence rate (ASIR), age-standardized prevalence rate (ASPR), and age-standardized DALY Rate (ASDR). Specifically, the ASIR rose from 48 per 100,000 in 1990 to 60 per 100,000 in 2021. Correspondingly, the ASPR increased from 362 per 100,000 in 1990 to 474 per 100,000 in 2021. Finally, the ASDR also showed an upward trend, climbing from 31 per 100,000 in 1990 to 41 per 100,000 in 2021. The AAPC of the ASIR, ASPR, and ASDR in China was 0.7434%, 0.8765%, and 0.8827%, respectively, significantly outpacing the global AAPC of 0.2204%, 0.2220%, and 0.2426%, respectively. The burden of psoriasis in China varied with age and gender, showing a trend of increasing and then decreasing ASIR, ASPR, and ASDR as age advanced. Women experienced lower incidence and prevalence rates of psoriasis than men. Over time, a delay in peak incidence was observed in both genders. The APC analyses revealed that psoriasis incidence initially increased and then declined with advancing age. Across all age groups, earlier birth cohorts had a relatively lower risk. Projections suggest that the incidence and prevalence of psoriasis in China will continue to rise over the next 15 years.

**Conclusion:**

Psoriasis poses a substantial public health challenge in China due to the country’s large and increasingly aging populace. Mitigating this burden requires a multifaceted approach, including precise epidemiological research, an enhanced understanding of its socioeconomic determinants, and the development of effective health policies.

## 1 Introduction

Psoriasis, a prevalent chronic papulosquamous skin condition, affects more than 125 million individuals worldwide (1). It is characterized by scattered or diffuse erythematous plaques covered with scales (2), leading to physical discomfort such as itching, pustules, and joint pain (3). Although not typically life-threatening, psoriasis significantly affects patients’ quality of life. In addition to psoriasis-associated discomfort or disability, patients with psoriasis endure a considerable financial burden, including direct medical costs, indirect expenses, and intangible costs (4). These challenges are compounded by reduced work productivity and varied degrees of prejudice and social stigma, which further diminish their standard of living (5, 6). The broader impact of psoriasis extends beyond individual suffering as the condition is a significant contributor to disability and illness. The health burden of disease is typically defined as the total cost of disease, disability, and/or premature death to the patient, the patient’s family, and society, encompassing factors such as loss of life, economic impact, and reduced quality of life (7). Traditionally, disease burden has been assessed using metrics such as incidence (the rate of new cases over a period) and prevalence (the total number of existing cases at a specific time and location). However, these measures are often limited in capturing the full scope of health impacts. To address this limitation, the Global Burden of Disease study (GBD study) evaluates disease burden using disability-adjusted life years (DALYs) (8), which incorporate both longevity and quality of life. This methodology has been widely adopted by numerous countries as a foundation for developing global disease assessments and measuring the effectiveness of health policies.

Current GBD studies on disease burden focus on macro-level assessments, analyzing disease burden at both global and regional scales. These studies have tracked psoriasis trends from 1990 to 2019, explored correlations with socioeconomic development, and projected future global burdens (9). However, this research largely overlooks the variations among countries and regions, failing to account for nation-specific characteristics and trends. The medical community placed significant focus on the psoriasis burden in China, the world’s most populous nation. Despite its global significance, comprehensive studies examining psoriasis progression in the Chinese population, alongside comparisons with global psoriasis trends remain scarce. Hence, this study provides a comprehensive analysis of the psoriasis burden in China from 1990 to 2021, comparing it with global trends using the latest GBD data. We performed join-point regression analysis and age-period-cohort (APC) modeling to explore variations in psoriasis across age groups, time periods, and birth cohorts in China. Additionally, we conducted in-depth analyses of variations in the psoriasis burden over 32 years, focusing on the impact of age and gender, and applied the autoregressive integrated moving average (ARIMA) model to project psoriasis trends for the next 15 years. We aim to equip policymakers with valuable insights into the psoriasis burden in China, facilitating the development of targeted prevention strategies and the efficient allocation of public health resources.

## 2 Methods

### 2.1 Data source

The data used in this study were derived from the GBD 2021 dataset, a comprehensive repository that includes incidence, prevalence, and mortality rates for over 300 diseases and injuries across 204 countries and territories, disaggregated by age and gender (10). This dataset was compiled from a wide range of sources, with a total of 56,604 inputs, including vital registration systems, cause-of-death extrapolations, population censuses, monitoring systems, research studies, and cancer registries (11).

For this study, data specific to psoriasis were obtained from the Global Health Data Exchange and its associated tools, accessible via the GBD Results Tool.^1^ Using this platform, we extracted data on incidence, prevalence, and DALYs for psoriasis in China and globally for the period 1990–2021. These indicators were used to assess the burden of psoriasis. As the 2021 GBD data is publicly available, this study did not require ethics committee approval. Furthermore, the study adhered to established guidelines for accurate and transparent reporting in health assessments.

### 2.2 Statistical analysis

We conducted an analysis of the incidence, prevalence, and DALYs for psoriasis in China and globally, focusing on their age-standardized rates (ASIR, ASPR, ASDR). Moreover, we explored crude incidence rates (CIR), crude prevalence rates (CPR), and crude DALY rates (CDR) for specific age groups, with all data sourced from the GBD database.

Using Join-point software (version 5.2.0), the average annual percentage change (AAPC) and its corresponding 95% uncertainty intervals (95% UI) were calculated to identify trends in disease burden (12, 13). The log-transformed age-standardized metrics were fitted to a regression model: ln(y) = *α* + *β*x + *ε*, where y represents the age-standardized metric, x denotes the calendar year, and ε is the error term. The AAPC was derived as 100 × (exp(β) - 1), with the 95% UI calculated from the model. Trends were categorized as increasing trends if the 95% UI of the AAPC estimate was greater than 0, decreasing if it was less than 0, and stable if equal to 0. Statistical analysis and visualization were performed using R statistical software (version 4.3.3) and Join-point software (version 5.2.0), with statistical significance set at *p* < 0.05.

The Age-Period-Cohort model (APC model) is a statistical framework widely used in social sciences, particularly for studying social change and human development. Its fundamental principle is to analyze the impact of these effects—age, period, and cohort—on a specific dependent variable within a unified framework. Due to the temporal confounding of these three effects, statistical methods are required to disentangle and quantify their individual contributions. We employed the APC model to evaluate how age, period, and cohort influence health outcomes. Age effects reflect biological and social aging processes that impact outcomes, period effects represent changes resulting from factors influencing all age groups simultaneously at specific time points, and cohort effects arise from shared experiences among individuals born in the same time frame. The APC model’s log-linear regression equation is as follows: log(Y_i_) = *μ* + α*age_i_ + β*period_i_ + γ*cohort_i_ + *ε*, where Y_i_ denotes the prevalence or mortality rate of psoriasis; α, β, and γ are coefficients for age, period, and cohort, respectively; μ denotes the intercept, and ε represents the residual. The combined effect of these factors was derived using an intrinsic estimator approach (14). For the model, data were divided into consecutive 5-year intervals from 1992 to 2021, excluding 1990–1991 because they deviated from the 5-year interval scheme. Ages were grouped into 5-year ranges (<5 years to >95 years), and 20 cohorts were defined, spanning birth years from 1897–1902 to 2012–2017. The reference group was the average age, period, and cohort (15, 16). Relative Risk (RR) values were calculated for each age, period, and cohort compared with the reference group as follows: [Exp(*α* - α_mean_), Exp(*β* - β_mean_), Exp(*γ* - γ_mean_)]. These values represent the independent risk.

The Autoregressive Integrated Moving Average Model (ARIMA Model) is a statistical model employed for the analysis and forecasting of time series data. The fundamental concept of the ARIMA model is to treat the data series formed by the predicted object over time as a random sequence. It uses a specific mathematical model to characterize the sequence based on the autocorrelation analysis of the time series. Once the model is established, it can predict future values from past and present values of the time series. We utilized the ARIMA model to forecast future trends in psoriasis burden. It consists of three components: autoregressive (AR), integrated (I), and moving average (MA). The model operates on the assumption that time-series data are time-dependent random variables with autocorrelation patterns, allowing for predictions of future values based on historical data. The model’s equation is as follows: Y_t_ = ϕ_1_Y_t-1_ + ϕ_2_Y_t-2_ + … + ϕ_p_Y_t-p_ + e_t_ - θ_1_e_t-1_ - … - θ_q_e_t-q_, where (ϕ_1_Y_t-1_ + ϕ_2_Y_t-2_ + … + ϕ_p_Y_t-p_ + e_t_) represents the AR coefficients; e_t_ - θ_1_e_t-1_ - … - θ_q_e_t-q_ denotes the MA coefficients; Y_t-p_ denotes the observed value at time (t - p); p and q are the orders of the AR and MA components, respectively; and e_t_ is the random error at time t (17). In this model, the time series should be a smooth random series with a mean of zero. To ensure stationarity, the time series data were stabilized using differencing before modeling. Subsequently, we used the auto.arima() function to select the optimal model based on the Akaike Information Criterion (AIC) (18). The analysis and visualization of ARIMA results were performed using the “forecast,” “tseries,” and “ggplot2” software packages in R version 4.3.3, with statistical significance determined at a two-tailed *p*-value threshold of <0.05.

## 3 Results

### 3.1 Overview of the psoriasis burden in China and globally

#### 3.1.1 Incidence of psoriasis in China and globally

The number of psoriasis instances in China rose from 530,265 (95% UI: 513,660–548,626) in 1990 to 1,012,635 (95% UI: 979,127–1,044,617) in 2021, representing a total growth of 90.97%. Simultaneously, on a global scale, the incident number increased from 2,852,676 (95% UI: 2,764,294–2,943,367) in 1990 to 5,099,418 (95% UI: 4,945,748–5,254,031) in 2021, reflecting a cumulative increase of 78.76%.The Age-Standardized Incidence Rate(ASIR) measures new disease cases per 100,000 people, adjusted for age. This adjustment is essential because the incidence of disease varies across different age groups. It standardizes comparisons among populations with different age structures, facilitating the identification of disease trends over time and across regions. In China, the ASIR increased from 48 (95% UI: 46–49) per 100,000 in 1990 to 60 (95% UI: 58–62) per 100,000 in 2021. Globally, ASIR increased from 57 (95% UI: 55–59) per 100,000 in 1990 to 62 (95% UI: 60–64) per 100,000 in 2021 (Table 1).

**Table 1:** All-age cases and age-standardized incidence, prevalence, and DALYs rates of psoriasis in China and globally in 1990 and 2021.

Location	Measure	1990		2021	
		All-ages cases	Age-standardized rates per 100,000 people	All-ages cases	Age-standardized rates per 100,000 people
China	Incidence	530,265 (513,660–548,626)	48 (46–49)	1,012,635 (979,127–1,044,617)	60 (58–62)
	Prevalence	3,921,863 (3,789,618–4,053,307)	362 (350–374)	8,453,045 (8,161,389–8,743,685)	474 (459–489)
	DALYs	343,657 (247,483–461,828)	31 (23–42)	728,553 (528,686–971,655)	41 (30–55)
Global	Incidence	2,852,676 (2,764,294–2,943,367)	57 (55–59)	5,099,418 (4,945,748–5,254,031)	62 (60–64)
	Prevalence	23,056,630 (22,317,288–23,805,091)	478 (462–493)	42,983,446 (41,654,457–44,313,231)	516 (500–532)
	DALYs	1,996,756 (1,441,303–2,670,809)	41 (30–55)	3,689,928 (2,684,040–4,917,113)	44 (32–59)

#### 3.1.2 Prevalence of psoriasis in China and globally

Regarding the prevalence rates, the number of psoriasis instances in China grew from 3,921,863 cases (95% UI: 3,789,618–4,053,307) in 1990 to 8,453,045 cases (95% UI: 8,161,389–8,743,685) in 2021, an aggregate growth of 115.54%. In the meantime, on the global scale, the prevalent number rose from 23,056,630 (95% UI: 22,317,288–23,805,091) in 1990 to 42,983,446 (95% UI: 41,654,457–44,313,231) in 2021, a cumulative increase of 86.42%. ASPR indicates the age-adjusted proportion of a population with a specific disease at a given time, reflecting the overall disease burden by accounting for both new and existing cases. It facilitates comparisons across populations with different age distributions, offering valuable insights into disease prevalence and its public health impact. In China, the ASPR increased from 362 (95% UI: 350–374) per 100,000 people in 1990 to 474 (95% UI: 459–489) per 100,000 people in 2021. Globally, overall ASPR increased from 478 (95% UI: 462–493) per 100,000 population in 1990 to 516 (95% UI: 500–532) per 100,000 population in 2021 (Table 1).

#### 3.1.3 DALYs of psoriasis in China and globally

A Disability-Adjusted Life Year (DALY) can be regarded as one “healthy” year lost due to disability or death caused by a certain reason. The sum of all DALYs in a population can be considered as the gap between the ideal state of healthy longevity and the current health status of the population, where no one is afflicted by diseases or disabilities. In other words, it represents the extent of disease burden. Globally, the number of DALYs for psoriasis was 1,996,756 (95% UI: 1,441,303–2,670,809) in 1990 and 3,689,928 (95% UI: 2,684,040–4,917,113) in 2021, representing an increase of 84.80% from 1990. Psoriasis-related DALYs in China increased by 112.00% between 1990 and 2021, from 343,657 (95% UI:247,483–461,828) in 1990 to 728,553 (95% UI:528,686–971,655) in 2021. Regarding the global ASDR, it increased from 41 (95% UI: 30–55) per 100,000 people in 1990 to 44 (95% UI: 32–59) per 100,000 people in 2021. In China, ASDR increased from 31 (95% UI: 23–42) per 100,000 people in 1990 to 41 (95% UI: 30–55) per 100,000 people in 2021 (Table 1).

#### 3.1.4 Global and Chinese trends in psoriasis burden

Between 1990 and 2021, the ASPR of psoriasis rose gradually both in China and globally. However, the increase was more pronounced in China, with the steepest rise occurring between 1995 and 2000. By contrast, the ASIR and ASDR for psoriasis in both China and globally remained relatively stable during the same period (Supplementary Figure 1).

### 3.2 Join-point regression analysis of the burden of psoriasis in China and globally

**Figure 1:**
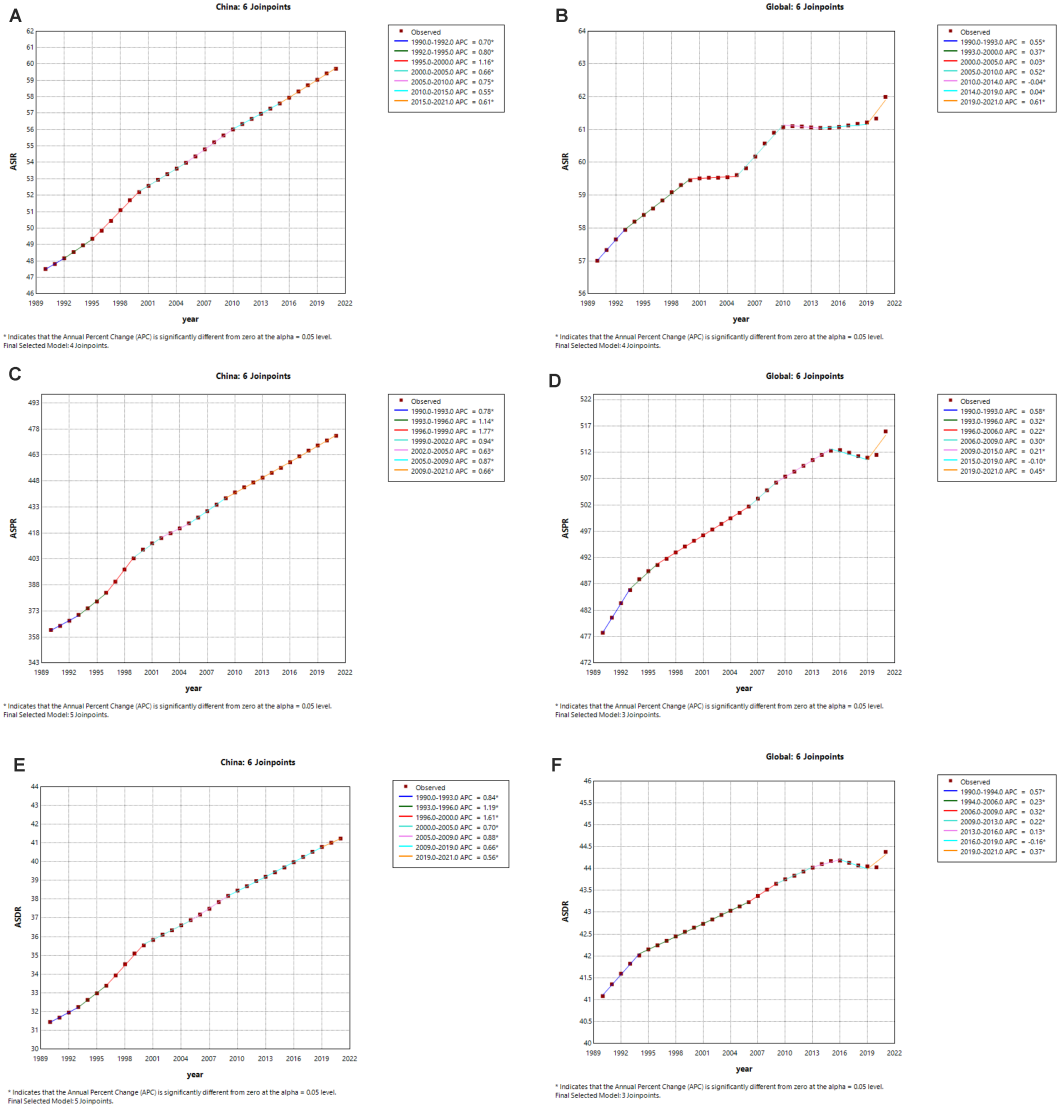
The APC of ASIR, ASPR and ASDR of psoriasis in China and Global from 1990 to 2021(*means *p*-values <0.05 and significant results). **(A,B)** ASIR; **(C,D)** ASPR; **(E,F)** ASDR.

The join-point regression analysis of ASIR, ASPR, and ASDR for psoriasis in China and globally from 1990 to 2021 is shown in Figure 1. According to these findings, from 1990 to 2021, the APCs of ASIR, ASPR, and ASDR of psoriasis in China exhibited an upward trend, especially during the period 1990–2000 (ASIR: 1995–2000 APC = 1.16, *p* < 0.05; ASPR: 1993–1996 APC = 1.14; 1996–1999 APC = 1.77, *p* < 0.05; ASDR: 1993–1996 APC = 1.19; 1996–2000 APC = 1.61, *p* < 0.05). Globally, the APCs for ASIR, ASPR, and ASDR in psoriasis have generally shown an increasing trend with slight fluctuations. ASIR decreased slightly from 2010 to 2014 (ASIR: 2010–2014 APC = −0.04, *p* < 0.05), and ASPR and ASDR decreased from 2015 to 2019 (ASPR: 2015–2019 APC = −0.10, *p* < 0.05; ASDR: 2016–2019 APC = −0.16, *p* < 0.05) and increased in the remaining years.

### 3.3 APC effects on psoriasis incidence

**Figure 2:**
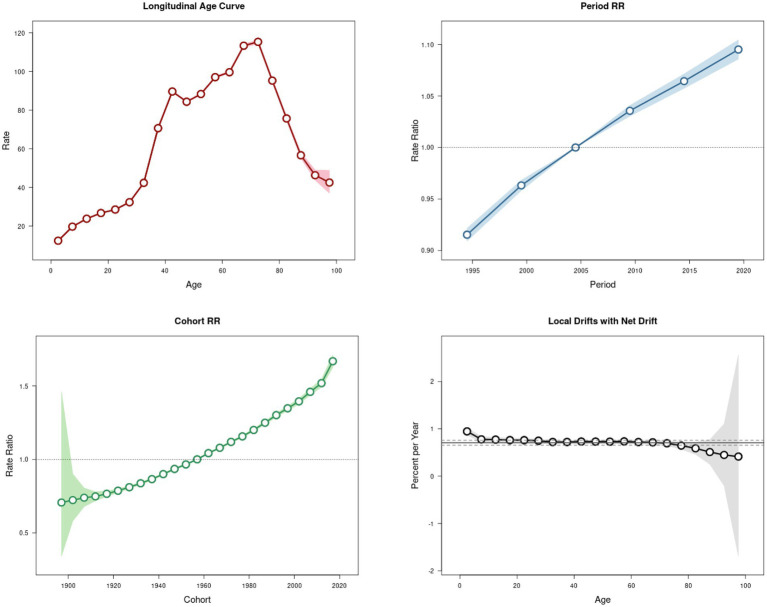
Trends of psoriasis incidence in China during 1992–2021.

Figure 2 presents the APC effect on psoriasis incidence. The APC analysis of psoriasis incidence reveals an overall increasing trend from birth to approximately 75 years of age, with the highest increase observed in the 32.5–37.5 age group. A slight decline occurs in the 42.5–47.5 age group, followed by a peak incidence at 72.5 years, after which the trend declines. After adjusting for the effects of age and cohort factors, the incidence demonstrated an increase over time, with RR values increasing from 0.915 (95% UI: 0.909–0.922) in 1995 to 1.095 (95% UI: 1.086–1.105) in 2020. An analysis of birth cohorts showed that early cohorts experienced lower psoriasis prevalence than late birth cohorts. The cohort risk was lower in early birth cohorts (RR_cohort (1897–1902)_ = 0.706, 95% UI: 0.342–1.460) and higher in more recent birth cohorts (RR_cohort (2012–2017)_ = 1.518, 95% UI: 1.492–1.546). Moreover, the local drift indices were consistently above 0 across all age groups, indicating an annual increase in psoriasis prevalence.

### 3.4 Burden of psoriasis among various age groups in China in 1990 and 2021

**Figure 3:**
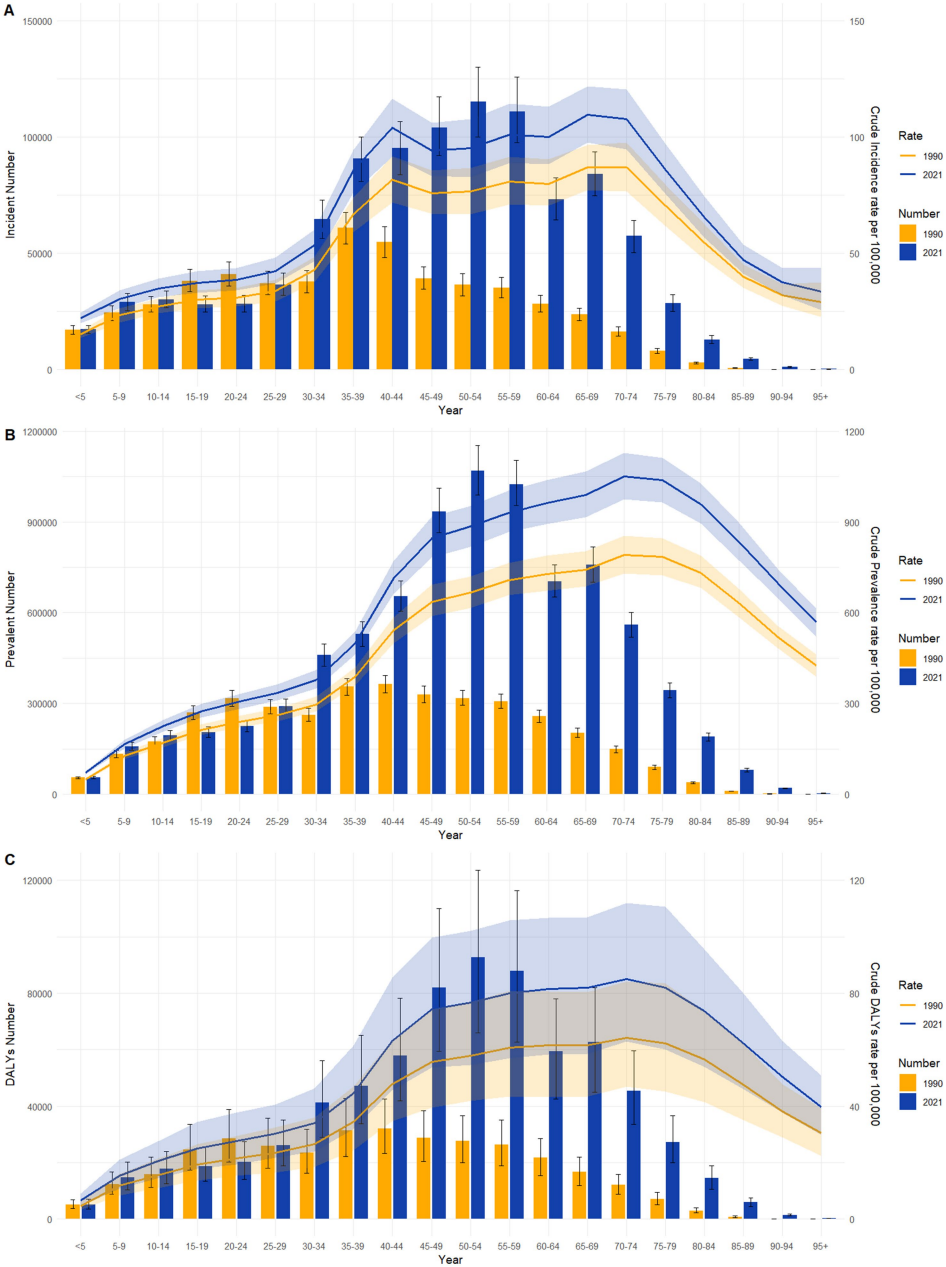
Comparative of the incidence, prevalence and DALYs counts, along with their crude rates, by age group in China from 1990 and 2021. **(A)** Incident cases and CIR; **(B)** Prevalent cases and CPR; **(C)** DALYs counts and CDR. Bar charts represent counts; lines represent crude rates.

Figure 3 compares the incidence, prevalence, and DALYs associated with psoriasis across different age groups in China for 1990 and 2021, as well as corresponding crude rates. According to the data on incidence, the 35–39 age group had the highest number of new psoriasis cases in 1990, whereas this shifted to the 50–54 age group in 2021. The crude incidence rates indicated two distinct peaks in psoriasis cases in 2019 and 2021 in China, observed in the 40–44 and 65–69 age groups. Crude prevalence rates exhibited a pattern of increase–decrease-a small increase in China from ages 0–29, a significant increase from ages 30–44, and a decline beyond age 75. DALYs peaked in the 40–44 age group in 1990 but shifted to the 50–54 age group in 2021, reflecting a backward shift in the peak burden over time.

### 3.5 Gender-based variations in psoriasis burden across age groups in China

**Figure 4:**
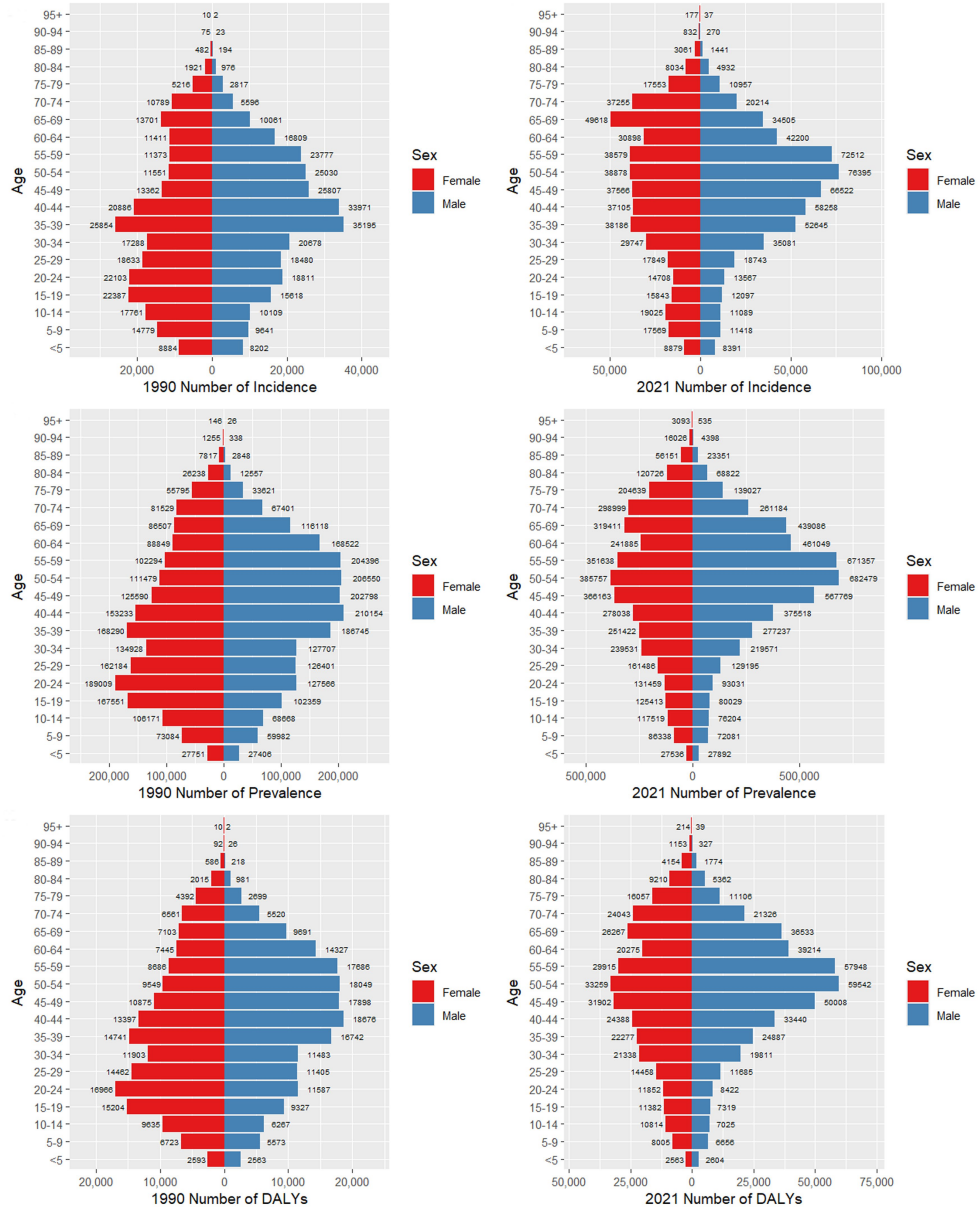
Comparison of the number of incidence, prevalence, and DALYs of psoriasis in males and females of different age groups in China in 1990 and 2021.

Figure 4 presents a detailed comparison of the incidence, prevalence, and DALYs for psoriasis among men and women across various age groups in China for the years 1990 and 2021. In 1990, the incident number peaked in the 35–39 age groups for both genders, whereas in 2021, the peak shifted to the 65–69 age group for women and the 50–54 age group for men. Notably, in 1990, the number of male cases was higher than that of female cases in the 30–64 age range, while females had a higher incidence in the other age groups. By contrast, by 2021, the age groups where males exhibited higher morbidity than females expanded to include the 25–64 age range. Trends in prevalence in 1990 showed a general upward trend followed by a downward trend for both genders, with the highest prevalence occurring in the 20–24 age group for women and the 40–44 age group for men. The peak prevalence for females occurred earlier than that for males. By 2021, both genders had their highest prevalent number in the 50–54 age group. This shift represents a delayed peak in women and a slight advancement in men compared to 1990. The DALYs exhibited a similar pattern, with a more pronounced and differentiated change between age groups in 2021 than in 1990.

The comparative analysis of the overall disease burden and ASIR of psoriasis in both male and female populations of all ages within China between 1990 and 2021 is presented in Supplementary Figure 2. The data indicates that in 1990, the ASIR of psoriasis for both men and women was at its lowest, with minimal gender disparity. As age increased, the ASIR rose steadily, and the gender difference also expanded over time. Moreover, the trends in ASPR and ASDR followed a similar trajectory to that of the ASIR.

### 3.6 Projected trends in psoriasis incidence and prevalence for the next 15 years

**Figure 5:**
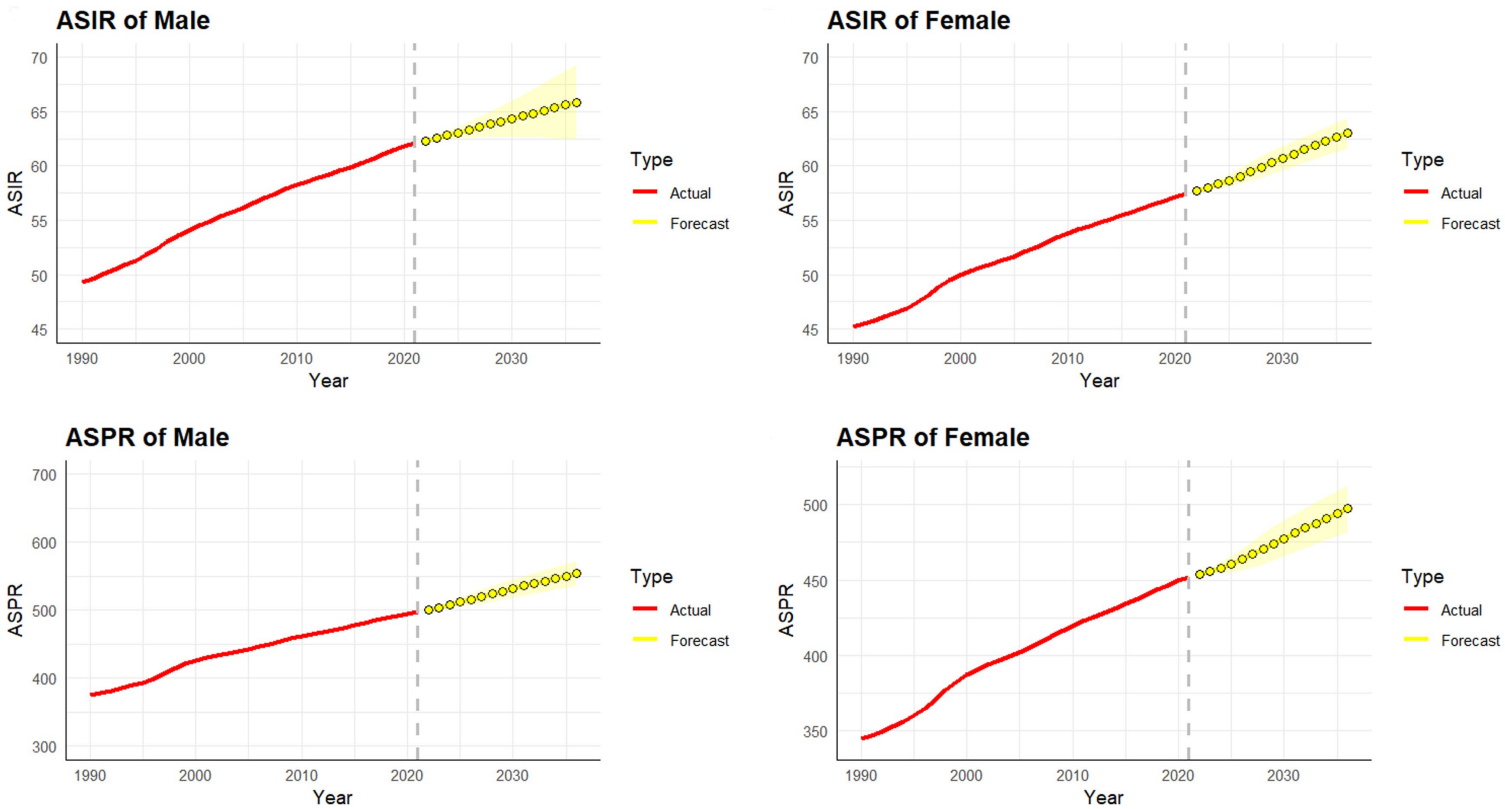
Predicted trends of psoriasis incidence and prevalence rate in China over the next 15 years (2022–2036). Red lines represent the true trend of psoriasis incidence and prevalence rate during 1990–2021; yellow dot lines and shaded regions represent the predicted trend and its 95% UI.

The ARIMA model was employed to forecast the incidence and prevalence of psoriasis in China for the next 15 years, stratified by gender. In terms of the ASIR, it is projected that for men, it will increase from 62.06 per 100,000 in 2021 to 65.85 per 100,000 in 2036, while for women, it will rise from 57.44 per 100,000 in 2021 to 62.99 per 100,000 in 2036. Regarding the ASPR, it is expected that for men, it will increase from 497.32 per 100,000 in 2021 to 554.17 per 100,000 in 2036, and for women, it will rise from 451.98 per 100,000 in 2021 to 497.08 per 100,000 in 2036. These projections indicate a steady increase in both incidence and prevalence of psoriasis among both genders in the coming years (Figure 5).

## 4 Discussion

This study provides a comprehensive evaluation of the incidence, prevalence, and DALYs associated with psoriasis in both China and globally over the past 32 years using the GBD 2021 database. We examined the variations in psoriasis disease burden across different age and gender groups, revealing significant trends in the disease’s impact. An upward trajectory in ASIR, ASPR, and ASDR for psoriasis was observed in both China and globally from 1990 to 2021. Our analysis highlighted that the incidence, prevalence, and DALYs associated with psoriasis are strongly correlated with the patient’s age, with a higher prevalence typically observed in middle-aged and older people. This is consistent with the findings of existing studies reporting that psoriasis often coexists with other conditions, such as cardiovascular disease, obesity, type II diabetes mellitus, dyslipidemia, nonalcoholic fatty liver disease, anxiety, depression, and inflammatory bowel disease (19, 20). These comorbidities are more prevalent in the middle-aged and older populations. Therefore, the age distribution of psoriasis onset is a crucial factor to consider in understanding the disease burden.

Between 1990 and 2021, China experienced rapid social and economic development, which contributed to significant increases in both the prevalence and incidence of psoriasis. In China, this trend was observed across all age groups, reflecting lifestyle changes, increased psychological stress, and improvements in diagnosis and public awareness of the disease (21). The period trend confirmed this increase in psoriasis burden across different age groups. Over the same period, the DALYs for psoriasis in China grew at an almost consistent rate, driven by the rise in the number of cases, longer life expectancy, and prolonged survival due to advancements in medical treatment. The GBD 2021 data suggested that the incidence of psoriasis is closely linked to age and that although the disease can develop in all age groups, the peaks of incidence were observed in the 35–39 and 65–69 age groups. This bimodal distribution of psoriasis incidence is consistent with global trends (22). Our current study also revealed that the incidence and prevalence of psoriasis in China were lower among women than among men. However, this finding warrants further investigation. Furthermore, research indicates that psoriasis tends to have a greater impact on the physical and psychosocial quality of life in women than in men, with women being more likely to experience associated conditions such as anxiety or depression (23, 24). Moreover, across regions worldwide and throughout various timeframes, women tend to have a longer lifespan than men, resulting in a prolonged duration of psoriasis-associated health burden for women (25). Additionally, women have a higher age of psoriasis onset than men (25, 26). Our study observed a trend of delayed onset, which may indicate increased longevity and improvements in overall population health.

According to the Socio-Demographic Index (SDI), countries are categorized into five levels of development: high, high-middle, middle, low-middle, and low. In 2017, China was categorized as a high-middle SDI country (26). Between 1990 and 2021, China’s SDI level increased significantly, and this rapid socioeconomic development coincided with a notable rise in the incidence of psoriasis, surpassing the global average. This suggests a strong association between psoriasis incidence and the SDI, reflecting that socioeconomic development may influence psoriasis rates by shaping lifestyle factors. Although economic development may not be the direct cause of psoriasis onset, it is it is associated with alterations in the natural environment and climate. For instance, certain infections can trigger the occurrence of psoriasis, while dry and cold climates may also induce or exacerbate the condition. Furthermore, with economic development, people’s lifestyles have changed. There has been an increase in life pressures, smoking, and alcohol consumption, all of which contribute to the onset and progression of psoriasis. In the past, due to the limitations of medical technology and information circulation, the detection rate of psoriasis may have been underestimated. Consequently, this rise may also reflect improvements in public health awareness and the diagnostic capabilities for psoriasis in China.

The Global Burden of Diseases (GBD), Injuries, and Risk Factors Study is the single largest and most comprehensive scientific endeavor ever conducted to quantify levels and trends in health. Researchers collect health data from hospitals, governments, surveys, and other databases worldwide. These data are then synthesized to generate the estimates that are published in the data visuals and publications. The GBD data are continuously updated and refined to reflect the latest research findings and disease trends, making them an essential tool for studying the global burden of disease. These data are extensively used in public health policymaking, disease prevention and control, medical research, and related fields. Although there are certain limitations—such as the accuracy and completeness of the data, which may be affected by factors like collection challenges, sample size, and discrepancies between countries and regions, potentially introducing uncertainties in global estimates—the reliability of GBD data has been widely recognized and validated.

Nevertheless, this study has several limitations. Firstly, the accuracy and comprehensiveness of the GBD statistics depend heavily on the quality and availability of data, including disease diagnosis and measurements of environmental risk factors over time. In regions with limited healthcare access and in economically disadvantaged populations, disease diagnosis may be incomplete, and psoriasis screenings may be insufficient, particularly in low-and middle-SDI areas globally (27). Some studies have indicated that self-reported prevalence of psoriasis often exceeds that of physician-reported prevalence (28), which suggests that the psoriasis burden could be underestimated in the data. Secondly, potential biases arising from improper classification and coding of diseases can undermine the reliability of the results. Furthermore, the evolution of diagnostic and detection methods, alongside variations in data collection practices and instruments, may introduce additional data biases. Additionally, GBD 2021’s disease burden indicators are limited to national and regional levels, which means that a more detailed analysis of psoriasis prevalence and burden across individual Chinese provinces was not feasible in this study. Overall, psoriasis represents a significant disease burden in China. Therefore, its epidemiological trends and their association with socioeconomic development must be continuously monitored. These insights will be crucial for guiding the development of public health policies, improving health awareness, and effectively addressing the progression of the disease across different age groups.

## Data Availability

The original contributions presented in the study are included in the article/Supplementary material, further inquiries can be directed to the corresponding author.

## Footnotes

^1^ https://vizhub.healthdata.org/gbd-results/

## Supplementary material

SUPPLEMENTARY FIGURE 1 Trend comparison of ASIR, ASPR, and ASDR of psoriasis in China and worldwide from 1990 to 2021.

SUPPLEMENTARY FIGURE 2 Comparison of full-age cases and age-standardized rates of incidence, prevalence and DALYs among men and women in China from 1990 to 2019. **(A)** Incident cases and ASIR; **(B)** Prevalent cases and ASPR; **(C)** DALYs counts and ASDR. Bar charts represent counts; lines represent age-standardized rates.
