# Supplementary figures and images for: Analysis and contrast of psoriasis disease burden trends in China and globally from 1990 to 2021
Source: Front Public Health. 2025 Mar 6;13:1541292. doi: 10.3389/fpubh.2025.1541292 (PMC11922690; doi:10.3389/fpubh.2025.1541292)

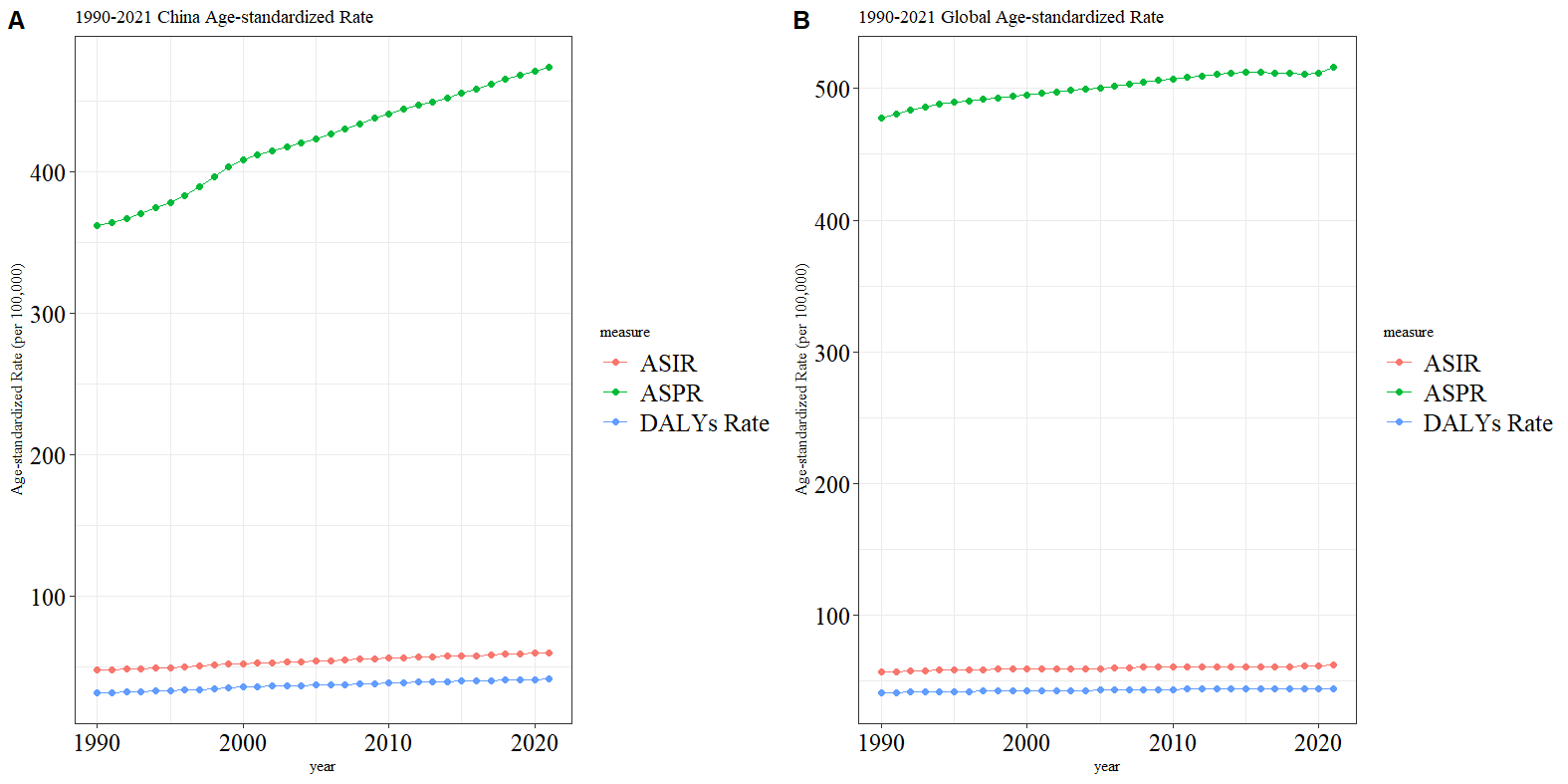

Supplement: SUPPLEMENTARY FIGURE 1 — Trend comparison of ASIR, ASPR, and ASDR of psoriasis in China and worldwide from 1990 to 2021. [file Image_1.tiff]

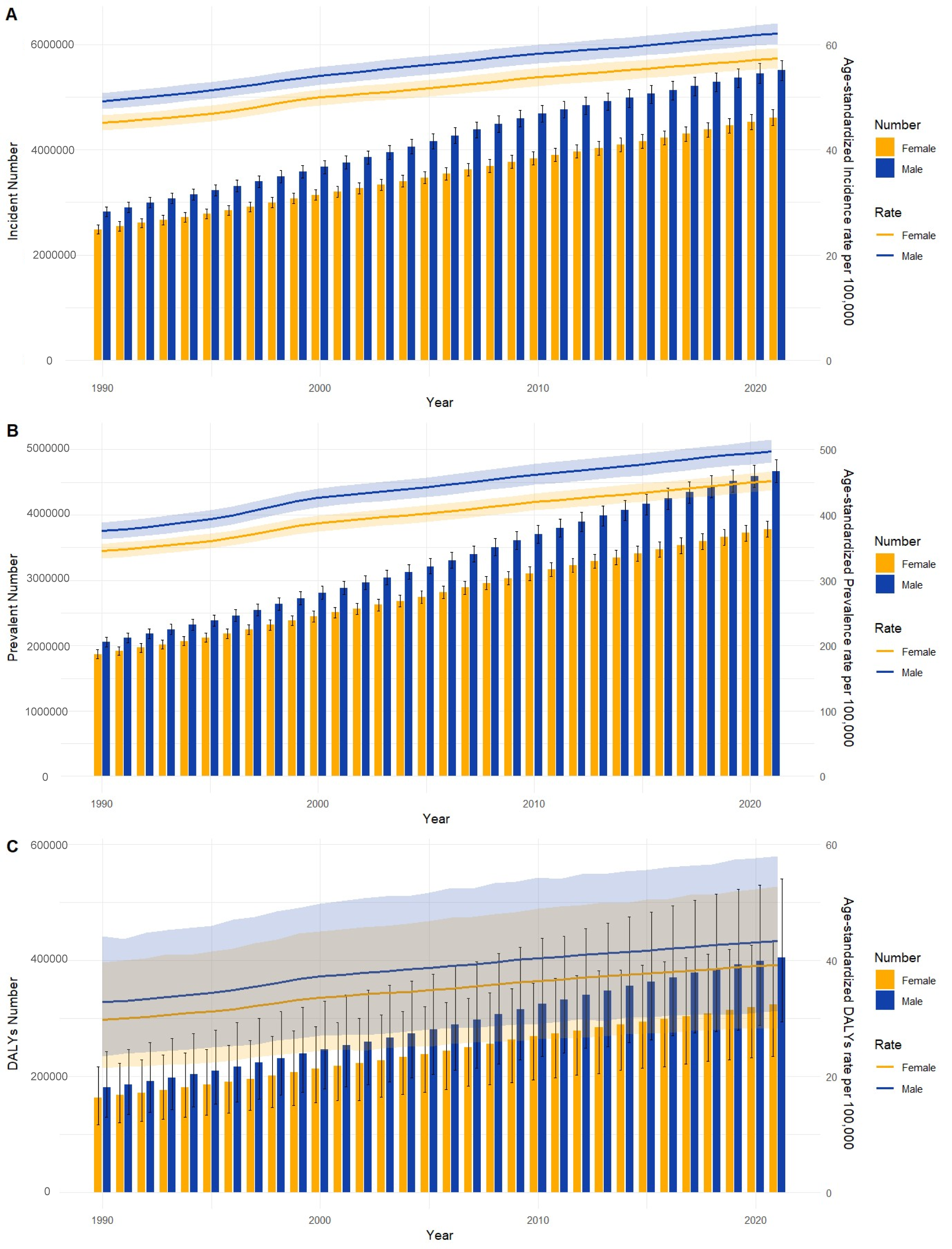

Supplement: SUPPLEMENTARY FIGURE 2 — Comparison of full-age cases and age-standardized rates of incidence, prevalence and DALYs among men and women in China from 1990 to 2019. (A) Incident cases and ASIR; (B) Prevalent cases and ASPR; (C) DALYs counts and ASDR. Bar charts represent counts; lines represent age-standardized rates. [file Image_2.tif]
